# *Schizochytrium* spp. Dietary Supplementation Modulates Immune-Oxidative Transcriptional Signatures in Monocytes and Neutrophils of Dairy Goats

**DOI:** 10.3390/antiox12020497

**Published:** 2023-02-16

**Authors:** Panagiota Kyriakaki, Alexandros Mavrommatis, Eleni Tsiplakou

**Affiliations:** Laboratory of Nutritional Physiology and Feeding, Department of Animal Science, School of Animal Biosciences, Agricultural University of Athens, Iera Odos 75, 11855 Athens, Greece

**Keywords:** microalgae, fatty acids, PUFA, DHA, ω6-DPA, eicosanoids, cytokines, antioxidants, oxidative stress, cyclooxygenase

## Abstract

The high propensity of dietary polyunsaturated fatty acids (PUFA) to oxidation can induce a cascade of cellular immune-oxidative imbalances. On the other hand, PUFA, namely docosapentaenoic acid (ω6-DPA) and docosahexaenoic acid (DHA) can exert immunomodulatory effects by suppressing a pro-inflammatory response. Thus, the objective of this study was to investigate the effect of dietary supplementation with *Schizochytrium* spp. levels, rich in both ω6-DPA and DHA on the transcriptional profiling of genes involved in oxidative homeostasis and innate immunity of dairy goats’ monocytes and neutrophils. Twenty-four dairy goats were divided into four homogeneous sub-groups; the diet of the control group (CON) had no *Schizochytrium* spp. while those of the treated groups were supplemented daily with 20 (ALG20), 40 (ALG40), and 60 (ALG60) g/goat/day. The mRNA levels of *MGST1* in neutrophils were downregulated (*p* = 0.037), while in monocytes, *SOD2* and *SOD3* were downregulated (*p* = 0.010 and *p* = 0.044, respectively) in ALG60 compared to the CON group. *GPX2* mRNA levels were downregulated (*p* = 0.036) in ALG20 and ALG60 compared to the CON group in neutrophils. *NOX1* was upregulated (*p* = 0.043) in the neutrophiles of ALG60-goats. *NOX2* was upregulated (*p* = 0.042) in the monocytes of ALG40-fed goats, while higher (*p* = 0.045) levels were also found in the ALG60 group in neutrophils. The mRNA levels of *COX2* were downregulated (*p* = 0.035) in monocytes of the ALG40 and ALG60 groups. The mRNA levels of *PTGER2* were also downregulated (*p* = 0.004) in monocytes of *Schizochytrium*-fed goats, while in neutrophils, significant downregulation (*p* = 0.024) was only found for ALG60 compared to the CON group. *ALOX5AP* mRNA levels were significantly decreased (*p* = 0.033) in ALG60 compared to the CON group in monocytes. *LTA4H* mRNA levels were increased (*p* = 0.015) in ALG60 compared to ALG20 and ALG40 groups in monocytes, while in neutrophils, a significant downregulation (*p* = 0.028) was observed in ALG20 compared to the CON group. The inclusion of more than 20 g *Schizochytrium* spp./day in goats’ diet induced imbalances in mechanisms that regulate the antioxidant system, while downregulated the expression of pro-inflammatory pathways in monocytes and neutrophils.

## 1. Introduction

Long-chain polyunsaturated fatty acids (LCPUFA), including docosapentaenoic (ω6-DPA) and docosahexaenoic (DHA) acids, are dietary fats with an array of health benefits. They are incorporated in many parts of the body, including cell membranes, and play a significant role in anti-inflammatory processes and in the viscosity of cell membranes [1,2,3]. In livestock, PUFA-rich feedstuffs are typically used in animal diets as a nutritional strategy to fortify animal products such as milk and meat, with PUFA designing functional foods fulfilling consumers’ current demands. In our previous studies, the dietary supplementation of marine microbe *Schizochytrium* spp., a unicellular eukaryote belonging to *Thraustochytriaceae* family rich in ω6-DPA and DHA, augmented ovine [4] and caprine [5,6] milk with DHA, DPA, and conjugated linoleic acid (CLA) resulting in a two-fold increase in milk PUFA content.

Beyond the beneficial and health-promoting properties of LCPUFA, their high propensity to oxidation can initiate a cascade of pro-oxidant incidences inducing oxidative stress [7]. Indeed, in our previous study investigating the effect of three inclusion levels of *Schizochytrium* spp. (20, 40, and 60 g/day) in goats’ diet, oxidative stress was induced as reflected by the higher activity of NADPH oxidase (NOX) in blood plasma and malondialdehyde (MDA) levels in both plasma and milk [8]. Similar results concerning oxidative stress were also observed in ewes supplemented with 20, 30, and 40 g *Schizochytrium* spp./day [4]. In the former experimental outcomes, a clear dose-dependent effect was observed, indicating that PUFA overload can deteriorate organism oxidative balance. However, it is still unclear if the endogenous antioxidant system can neutralize the reactive oxygen species (ROS) formed during oxidative burst or if these unstable metabolites (ROS) can disrupt the organism’s antioxidant defense since the outcomes regarding antioxidant enzymes activities were not fully consistent amongst the previous studies [4,6,8]. Additionally, ROS overproduction as a consequence of lipid peroxidation can also activate the nuclear factor-κB (NF-κB) translocation to the nucleus initiating the production of pro-inflammatory mediators resulting in a severe cytokine storm [9]. Hence, it is of utmost importance to assess the effect of dietary LCPUFA on the immune-oxidative interplay of neutrophils and monocytes since both cells activation via the innate immune system lead to ROS-production mainly via NADPH oxidase and vice versa [10].

Focusing on the impact of LCPUFA (eicosapentaenoic acid (EPA), ω6-DPA, and DHA) on immune regulation, they can efficiently substitute arachidonic acid (ARA) in the membranes of white blood cells, resulting in the production of eicosanoids with diverse pro-inflammatory and anti-inflammatory potential compared with those produced with ARA as precursor [11]. In our previous work, the inclusion of *Schizochytrium* spp. levels (20, 40, and 60 g/day) in goats’ diets downregulated the mRNA abundance of pro-inflammatory cytokines (*IFNG*, *IL1B*, *IL2*, *IL8*, *TNF*) and chemokines (*CCL5* and *CXCL16*) through the inhibition of the TLR4-MYD88-MAPK pathway in monocytes [12]. Based on this previous set of evidence, we hypothesized that the inclusion of DHA and ω6-DPA fatty acids in goats’ diet affected the synthesis of eicosanoids, which in turn promoted the production of resolvins and protectins which are involved in either the inhibition of NF-κB translocation or the activation of peroxisome proliferator-activated receptor-gamma (PPARγ) resulting in the downregulation of pro-inflammatory cytokines [13,14]. However, this hypothesis was not entirely investigated in our previous, preliminary study in which genes that regulated the immunity through fatty acids metabolism were not assessed.

Considering the aforementioned issues, the main objective of this study was to investigate the effect of dietary inclusion levels of ω6-DPA- and DHA-rich microalgae *Schizochytrium* spp. on the expression of genes involved in the oxidative balance in monocytes and neutrophils of goats. Furthermore, this study aimed to validate if the suppression of a pro-inflammatory response induced by PUFA inclusion in goats’ diet was attributed to alterations in the eicosanoid pathways and to inspect the immune-oxidative interplay orchestrated by NADPH oxidase.

## 2. Materials and Methods

### 2.1. Animal Trial 

This work followed the analytical approach introduced in previously published studies [5,8,12]. Twenty-four, three-to-four-year-old dairy goats at mid–late lactation (150 ± 10 days in milk) were clustered into four sub-groups (n = 6) according to their fat (4%)-corrected milk yield and body weight. The control group (CON) received a basal diet containing alfalfa hay (1 kg) and concentrate (1 kg/goat/day). The ALG20, ALG40, and ALG60 groups were offered the same diet supplemented with 20, 40, and 60 g *Schizochytrium* spp./goat/day included in the concentrate, respectively. *Schizochytrium* spp. was traded as a commercial product named DHAgold (DSM Human Nutrition & Health, Heerlen, The Netherlands). The chemical composition and fatty acid profile of the feedstuffs and *Schizochytrium* spp. are presented in Table S1. The trial lasted for 74 days; the first two weeks were preceded as an adaptational period. The duration of the experimental trial aimed to unveil any adaptational response to the diets while simultaneously minimizing the use of animals as experimental models. Further information about the ration design, microalgae inclusion, and chemical composition of the feeds was previously reported by Mavrommatis et al. [8].

### 2.2. Blood Collection, Cells Isolation, RNA Extraction, and cDNA Synthesis

Blood samples (10 mL) were taken from the jugular vein into 17 units/mL heparin-containing tubes (BD Vacutainer, Plymouth, UK) on the 20th, 40th, and 60th day from the beginning of the main experimental period for cell isolation. Samples were immediately transferred to the laboratory for monocytes and neutrophils isolation. Cells were isolated using Histopaque density gradient (Sigma-Aldrich Co., Burlington, MA, USA) after blood dilution (1:1 *v*/*v*) with Hanks Balanced Salt Solution (Sigma-Aldrich Co., Burlington, MA, USA). After centrifugation at 400× *g* for 40 min at 4 °C, monocytes were suspended above the Histopaque layer while neutrophils were precipitated within the red cells. Monocytes were collected and transferred into a new 50 mL-Falcon tube and both cell types were washed several times with phosphate buffer saline and 0.9% NaCl solution. After the cells’ isolation, they were resuspended in RPMI medium and counted. Total RNA was extracted from approximately 5 × 10^6^ cells using peqGOLD TriFast (VWR, International) according to the manufacturer’s instructions. A total of 5000 ng of RNA were treated with two units Turbo DNAse (Invitrogen, CA, USA) at 37 °C for 1 h. After verification for DNA contamination absence using agarose gel [12], RNA was precipitated and eluted with RNAse-free water. Then, 500 ng of pure RNA was reversely transcribed with the Prime-Script First Strand cDNA Synthesis Kit (Takara Japan), according to the manufacturer’s instructions using a mix of random hexamers and oligo-dT primers. 

### 2.3. Primer Design and Real-Time Quantitative PCR

Primers were designed according to their coding sequence of *Capra hircus* using Primer designing tool—NCBI—NIH (Table 1). Relative expression levels of mRNA for target genes were quantified with a step one real-time PCR system (Applied Biosystems, Foster City, CA, USA). PCR cycling started at 95 °C for 15 min, followed by 40 cycles of 95 °C for 15 s and 60 °C to 62 °C for 1 min (Table 1). Primers’ specificity and formation of primer dimers were also monitored via melt curve analysis and electrophoresis gel of the PCR products. The expression levels of glyceraldehyde-3-phosphate dehydrogenase (*GAPDH*) and tyrosine 3-monooxygenase/tryptophan 5-monooxygenase activation protein zeta (*YWHAZ*) were used as housekeeping genes to normalize cDNA templates [15]. The relative expression levels of the target genes were calculated as previously described [12].

### 2.4. Statistics

The data were analyzed using the SPSS. IBM (v. 26.0) and are presented as means and standard error of means (SEM) in Tables S2 and S3. Dietary group effects were tested using a general linear model for repeated measures analysis of variance with dietary groups (D = CON, ALG20, ALG40, ALG60) as a fixed factor and the sampling times (S = 20th, 40th, and 60th experimental day) as the repeated measure and their interactions (D × S). 

**Figure 1:**
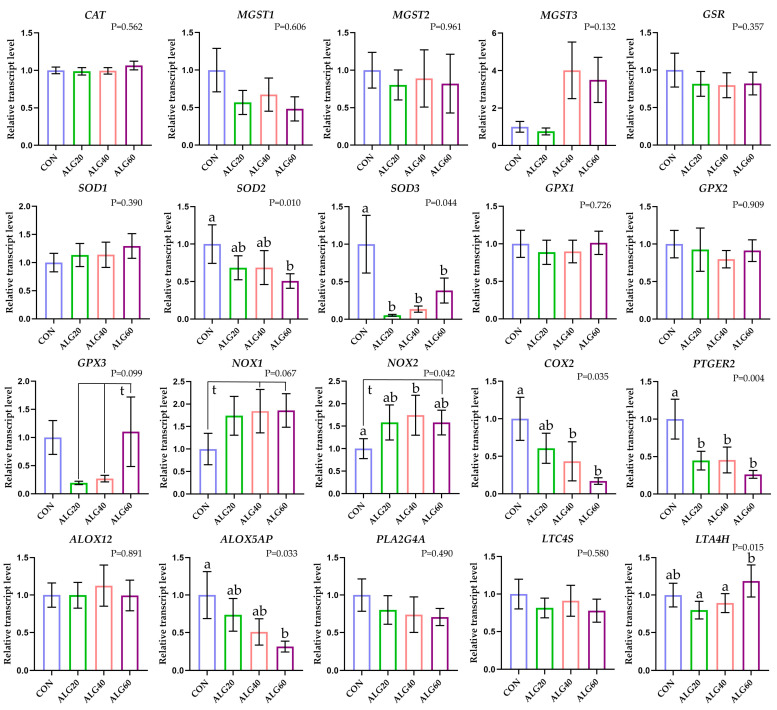
Mean and standard error (SE) of relative transcript levels as fold change of several anti- and pro-oxidant-related and immune-related gene expressions in monocytes of goats fed the four experimental diets (CON, ALG20, ALG40, and ALG60) at three sampling times (20th, 40th and 60th). Different superscript (a, b) between dietary treatments differ significantly (*p* ≤ 0.05) while, t; trend refers to *p* < 0.10.

**Figure 2:**
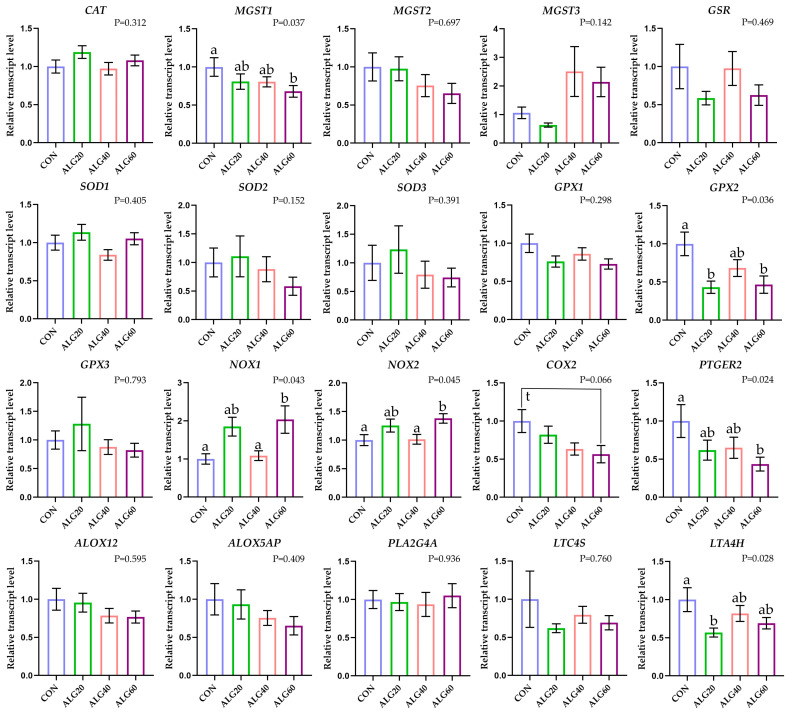
Mean and standard error (SE) of relative transcript levels as fold change of several anti- and pro-oxidant-related and immune-related gene expressions in neutrophils of goats fed the four experimental diets (CON, ALG20, ALG40, and ALG60) at three sampling times (20th, 40th and 60th). Different superscript (a, b) between dietary treatments differ significantly (*p* ≤ 0.05) while, t; trend refers to *p* < 0.10.

Post hoc analysis was executed using the LSD test while the significance threshold was set at 5%. GraphPad Prism 8.0 was used to depict gene expression in bars as fold changes between the control group and treatments. Error bars represent the standard errors (SE) and different superscript letters signify statistical differences emerging through repeated measure analysis (Figure 1 and Figure 2).

**Figure 3:**
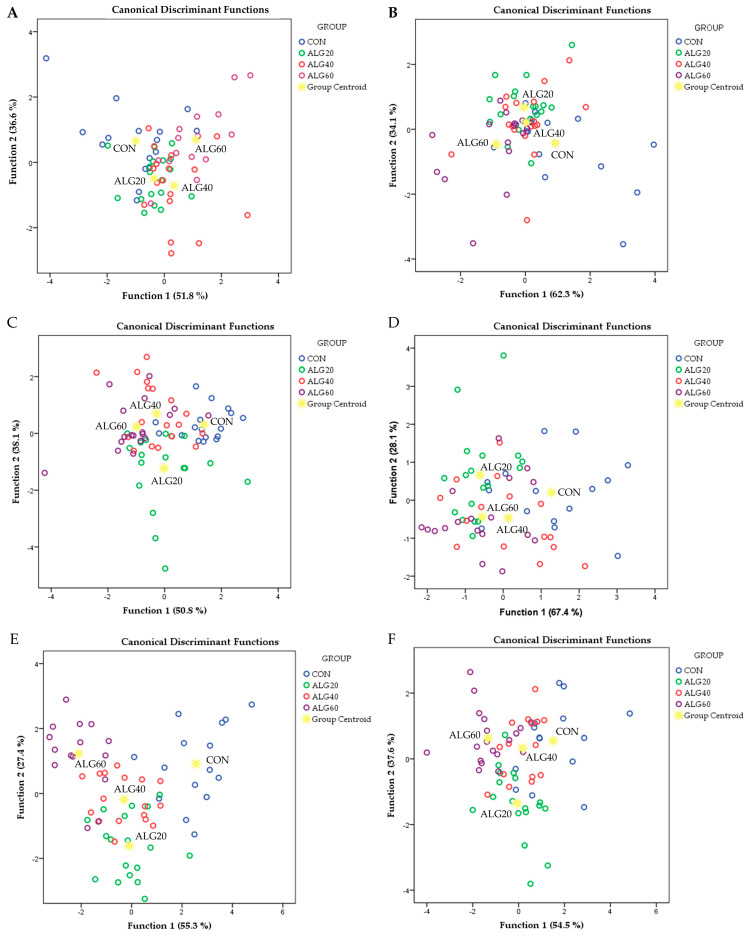
Discriminant plots separating the four dietary treatments (CON, ALG20, ALG40 and ALG60) according to (**A**) monocytes antioxidants and pro-oxidants genes, (**B**) monocytes immune-regulation genes (**C**) neutrophils antioxidants and pro-oxidants genes, (**D**) neutrophils immune-regulation genes, (**E**) monocytes and neutrophils antioxidants and pro-oxidants genes, and (**F**) monocytes and neutrophils immune-regulation genes.

Discriminant analyses were also applied to pooled data to assess those variables capable of distinguishing and classifying samples among the four dietary groups using Wilk’s lambda (λ) criterion (Figure 3). Variables for monocytes, neutrophils, and monocytes and neutrophils’ gene expression were entered (independent together) to develop six district models to discriminate the seventy-two samples of each. 

## 3. Results

### 3.1. Feed Consumption and Animal Performance

In the ALG60 group, a significant decrease (*p* < 0.001) was observed in dry matter intake for the concentrate, which led to a lower (40 g) than planned intake (60 g) of *Schizochytrium* spp. [5]. Despite the reduction in daily feed intake by ALG60 goats, their milk yield and body weight were not affected significantly (*p* > 0.05). Data about animal and milk performance are available in Mavrommatis and Tsiplakou’s work [5].

### 3.2. Effect of Schizochytrium spp. on Anti- and Pro-Oxidant-Related Gene Expressions of Monocytes and Neutrophils

*MGST3* mRNA abundance was numerically increased in ALG40 and ALG60 neutrophils and monocytes (*p* = 0.142 and *p* = 0.132 respectively); however, the fluctuations were not significant (*p* > 0.05) due to high standard errors (Figure 1 and Figure 2). The relative transcript levels of *MGST1* were significantly downregulated (*p* = 0.037) in ALG60 compared to the CON group in neutrophils (Figure 2). The relative transcript levels of *SOD2* were significantly downregulated (*p* = 0.010) in ALG60 compared to the CON group, while *SOD3* was downregulated (*p* = 0.044) in all *Schizochytrium*-fed goats’ monocytes (Figure 1). *GPX2* mRNA abundance was significantly downregulated (*p* = 0.036) in ALG20 and ALG60 compared to the CON group in neutrophils (Figure 1). *NOX1* mRNA abundance tended to increase (*p* = 0.067) in ALG40 and ALG60 groups compared to CON in monocytes, while its expression was significantly upregulated (*p* = 0.043) in the ALG60 group compared to CON and ALG40 goats in neutrophils. *NOX2* relative expression was upregulated (*p* = 0.042) in ALG40-fed goats monocytes, while significantly higher (*p* = 0.045) transcript levels were found in the ALG60 group of neutrophils compared to the CON group.

### 3.3. Effect of Schizochytrium spp. on Immune-Related Gene Expressions of Monocytes and Neutrophils

The relative transcript levels of *COX2* were downregulated (*p* = 0.035) in monocytes of the ALG40 and ALG60 groups (Figure 1), while in neutrophils, there was a tendency (*p* = 0.066) for decrease only in ALG60 compared to the CON groups (Figure 2). Additionally, *COX2* relative expression in monocytes showed a significant interaction (*p* = 0.038) between the dietary group and sampling time since its expression was decreased by *Schyzochytrium* spp. Supplementation, while a significant peak was observed during the second sampling time (Table S2). Downstream, the mRNA levels of *PTGER2* were also downregulated (*p* = 0.004) in monocytes of *Schizochytrium*-fed goats, while in neutrophils, significant downregulation (*p* = 0.024) was only found for ALG60 compared to the CON group. Moreover, *PTGER2* relative expression in monocytes showed a significant interaction (*p* = 0.001) between the dietary group and sampling time since its expression was decreased by *Schyzochytrium* spp. Supplementation, while a significant peak was observed during the second sampling time (Table S2). *ALOX5AP* relative transcript levels were significantly decreased (*p* = 0.033) in ALG60 compared to the CON group in monocytes. Furthermore, *ALOX5AP* relative expression in monocytes showed a significant interaction (*p* = 0.046) between the dietary group and sampling time since its expression was decreased by *Schyzochytrium* spp. Supplementation, while a significant peak was observed during the second sampling time (Table S2). *LTA4H* mRNA levels were increased (*p* = 0.015) in ALG60 compared to ALG20 and ALG40 groups in monocytes, while in neutrophils, a significant downregulation (*p* = 0.028) was observed in ALG20 compared to the CON group.

### 3.4. Holistic Statistical Analyses

Discriminant analyses were performed on the pooled data of monocytes and neutrophils’ gene expression in order to investigate if the samples can be distinguished according to the dietary experimental group (CON, ALG20, ALG40, and ALG60) (Figure 3). Starting from the monocytes’ antioxidants and pro-oxidants gene expressions (*CAT*, *MGST1*, *MGST2*, *MGST3*, *GSR*, *SOD1*, *SOD2*, *SOD3*, *GPX1*, *GPX2*, *GPX3*, *NOX1*, *NOX2*, and *NOX3*; Figure 3A), the percentage of the samples that were classified into the correct group, according to the dietary treatment, was 62.1%. Wilks’ lambda was observed at 0.377 for Function 1 (*p* = 0.045) and 0.611 for Function 2 (*p* = 0.267) and the relative expression of *NOX1*, *SOD1*, and *SOD2* were the variables that contributed the most. In neutrophils (Figure 3C), the percentage of the samples that were classified into the correct group, according to the dietary treatment, was 60.6%. Wilks’ lambda was observed at 0.306 for Function 1 (*p* = 0.001) and 0.542 for Function 2 (*p* = 0.038) and the relative expression of *GPX2*, *MGST1*, *GPX1*, and *MGST2* were the variables that contributed the most. In monocytes’ immune-related gene expressions (*COX2*, *PTGER2*, *ALOX12*, *ALOX5AP; PLA2G4A*, *LTC4S*, and *LTA4H*; Figure 3B), the percentage of the samples that were classified into the correct group, according to the dietary treatment, was 50.0%. Wilks’ lambda was observed at 0.545 for Function 1 (*p* = 0.008) and 0.785 for Function 2 (*p* = 0.200) and the relative expression of *PTGER2* was the variable that contributed the most. Additionally, in the immune-related gene expressions of neutrophils, a low percentage of correct classification was found (59.1 %). Wilks’ lambda was observed at 0.484 for Function 1 (*p* = 0.003) and 0.771 for Function 2 (*p* = 0.217) and the relative expression of *LTA4H*, *PTGER2*, and *LTC4S* were the variables that contributed the most (Figure 3D). Although the significance levels of function 1 were statistically significant in all four discriminant models (Figure 3A–D), the high values of Wilks’ lambda did not indicate a clear discrimination. However, by combining the variables of both monocytes’ and neutrophils’ gene expressions, higher levels of correct group classification and lower Wilks’ lambda values were achieved. More specifically, in monocytes’ and neutrophils’ antioxidants and pro-oxidants gene expressions (Figure 3E), the percentage of the samples that were classified into the correct group, according to the dietary treatment, reached 84.6%. Wilks’ lambda was observed at 0.062 for Function 1 (*p* < 0.001) and 0.230 for Function 2 (*p* = 0.023) and the relative expression of *GPX2* (neutrophils), *GPX1* (neutrophils), *MGST1* (neutrophils), *NOX1* (monocytes), *NOX2* (monocytes), *MGST1* (monocytes), *SOD1* (monocytes) and *SOD2* (monocytes) were the variables that contributed the most. Regarding the immune-related gene expressions of both monocytes and neutrophils, the percentage of the samples that were classified into the correct group, according to the dietary treatment, was 66.7 % (Figure 3F). Wilks’ lambda was observed at 0.239 for Function 1 (*p* < 0.001) and 0.496 for Function 2 (*p* = 0.046) and the relative expression of *PTGER2* (neutrophils), *PTGER2* (monocytes), *COX* (monocytes), *ALOX5AP* (monocytes), *LTC4S* (neutrophils), *ALOX12* (neutrophils), *PLA2G4* (monocytes) and *PLA2G4* (neutrophils) were the variables that contributed the most.

## 4. Discussion

The intensification of ruminants’ productive systems frequently exposes animals to a low-grade inflammation condition due to high energy and nutrient demands arising from high genetic merit and productivity [16,17]. A chronic form of low-grade inflammation can impair both animals’ productivity and health, compromising farmers’ revenue. This study, in combination with the outcomes of our previous work [12], indicates the potential of ω6-DPA and DHA fatty acids as feed additives in suppressing goats’ low-grade inflammation by inhibiting pro-inflammatory mediators. For this reason, our study focused on the assessment of immune-related transcriptional signatures at cells’ native condition without performing any challenging stimulus.

### 4.1. The Effect of Dietary Supplementation with Schizochytrium spp. on Pro- and Anti-Oxidant Gene Expressions

Although less individual significant alterations in the expression of genes related to antioxidants and pro-oxidants mechanisms compared to those regulating the immune system were found, the discriminant analyses indicated that *Schizochytrium* spp., rich in ω6-DPA and DHA, affected the antioxidant-pro-oxidant transcriptional profiling more firmly based on studied genes. The imbalances in oxidative biomarkers at transcriptional level in our study may be initiated by the influence of specific fatty acids contained in *Schizochytrium* spp. on NADPH oxidase (NOX) activity. Indeed, the mRNA levels of *NOX1* and *NOX2* were upregulated in both monocytes and neutrophils by the inclusion of high *Schizochytrium* spp. levels in goats’ diet. This hypothesis is further supported by the results of our previous study, where the inclusion of *Schizochytrium* spp. in goats’ diet increased the NOX enzymatic activity in blood plasma [8]. The biomass of *Schizochytrium* spp. further to its high proportion of PUFA such as DPA and DHA also contains a prominent amount of palmitic acid (PA; 13% of total fatty acids). In the study of Lambertucci et al. [18], PA increased NADPH oxidase (NOX) activity in rats’ cells, resulting in higher production of superoxide anion. Once the pro-oxidants incidences are started, PUFA are highly oxidized by ROS; thus, plenty of lipid peroxidation byproducts are formed [19]. Indeed, the inclusion of more than 20 g *Schizochytrium* spp. in goats’ [6,8] and ewes’ [4] diets increases the concentration of MDA in both their blood and milk. MDA is considered one of the most accurate indicators for lipids’ peroxidation [20]. Manea et al. [21] reported that compounds that accumulated from lipid peroxidation such as 4-Hydroxynonenal (4-HNE) and beta-unsaturated hydroxyalkenal can increase the NOX activity in human’s muscles cells. Thus, it could be assumed that the dietary fortification with PA and LCPUFA (ω6-DPA and DHA) at levels more than 30 mg and 70 mg/kg BW in small ruminants, respectively, can generate a vicious cycle of ROS overproduction through NOX activation, inducing lipid peroxidation.

SOD is the first defense line against oxidative stress, protecting the cells from damage by detoxifying the superoxide anion [22]. SOD catalyzes the dismutation of superoxide (O_2_^−^) into oxygen and hydrogen peroxide. There are three forms of SOD present in mammals: cytoplasmic superoxide dismutase (SOD1), mitochondrial superoxide dismutase (SOD2), and extracellular superoxide dismutase (SOD3) [23]. In this study, *SOD2* and *SOD3* relative expressions were downregulated in medium and high *Schizochytrium*-fed goats’ monocytes. However, the contribution of *SOD3* in RNA extracted from monocytes and neutrophils was expected to be negligible as regards the extracellular isoform. Indeed, in both cells, the mRNA levels of *SOD3* were close to the detection threshold (Tables S2 and S3). Nevertheless, a significant increase in SOD activity in the blood plasma of ALG40 and ALG60 goats has been observed [8], which can be partially attributed to the low correlation (up to 40%) between mRNA and protein levels [24].

CAT and GSH-Px are the second defense line against oxidative stress by catalyzing the conversion of hydrogen peroxide to water and oxygen as well as catalyzing the reduction of peroxide radicals to alcohols and oxygen. Similar to our observations about CAT activity in blood plasma in our previous work [8], monocytes and neutrophils’ *CAT* mRNA levels were not affected. On the other hand, neutrophils *GPX2* was significantly downregulated in ALG20 and ALG60 groups, while plasma GSH-Px activity was not affected [8]. A possible mechanism underlying *GPX* downregulation may be attributed to the higher formation of superoxide anions through NOX activation and, subsequently, the production of H_2_O_2_, which may result in inhibitory feedback on GSH-Px activity. More specifically, it was previously found that a high concentration of H_2_O_2_ inhibits the activity of GSH-Px [25]. Microsomal glutathione S-transferase 1 (*MGST1*) was also downregulated in neutrophils of *Schizochytrium*-fed goats. In agreement with the study by Rudkowska et al. [26], the dietary supplementation with n-3 PUFA decreased the expression of *MGST1* (17%). However, in the study by Rudkowska et al. [26], a significant downregulation was also found in the *TNFA* (37%), similar with that observed in our previous study [12].

### 4.2. The Effect of Dietary Supplementation with Schizochytrium spp. on Immune-Related Gene Expressions

Although the results of our previous work [12], in combination with the transcriptional profiling of *NOX* genes and the antioxidant gene regulators, indicate that PUFA inclusion and foremost in the higher supplementation levels disrupts the oxidative balance of goat organisms; interestingly, these changes did not trigger the pro-inflammatory response. In addition, a significant downregulation in the TLR4 pathway was found in monocytes of *Schizochytrium*-fed goats [12]. These findings could be attributed to two principal modes of action. The first one involves the natural antioxidant compounds contained in *Schizochytrium* biomass, namely carotenoids and phenolic compounds [27], which might be capable of neutralizing ROS produced by NADPH oxidase before they disturb other cells and gene networks initiating pro-inflammatory signals [28]. The previous assumption cannot be fully supported since MDA was significantly increased in blood of high-level *Schizochytrium*-fed goats [8]. The second and more conceivable one is related to the higher manipulation impact of ω6-DPA and DHA on the production of eicosanoids and, consequently, the formation of cytokines. The downregulation in the *COX2* and *PTGER2* genes in both monocytes and neutrophils of goats further points to a severe orchestration role of LCPUFA on pro-inflammatory mediators, supporting this second assumption. Taking into account the aforementioned findings, it can be assumed that two different modes of action might explain the outcomes of our work.

Dietary LCPUFA are incorporated into immune cell membranes, while EPA, DPA, and DHA substitute ARA. Notably, fish oil (rich in EPA and DHA) dietary inclusion in human and animal models decreased the proportion of ARA in immune cell membranes, while the concentrations of EPA and DHA were increased [2]. These LCPUFAs are detached from the phospholipid membrane through the action of A2 phospholipase (PLA2G4A) [2]. In our study, the mRNA levels of *PLA2G4A* were not altered by *Schizochytrium* spp. inclusion levels in goats’ diet, suggesting that the utilization efficiency of LCPUFA for eicosanoids production was not affected. However, in a previous study carried out by Smesny et al. [29], ω3 compared to ω6 PUFA dietary supplementation in humans decreased the activity of A2 phospholipase.

Pro-resolving lipid mediators are biosynthesized by the sequential actions of 15-LOX (ALOX15), 12-LOX (ALOX12), and 5-LOX (ALOX5) on DHA, DPA, and ARA, while the potent chemoattractant and pro-inflammatory mediator LTB4 is synthesized from ARA by 5-LOX (ALOX5) acting with 5-LOX activating protein (ALOX5AP), followed by LTA4 hydrolase (LTA4H) [30]. *ALOX5AP* was significantly downregulated in monocytes of ALG60-fed goats, while in neutrophils, *LTA4H* was decreased in ALG20-fed goats. The inhibition of the 5-LOX pathway could be explained by the lower availability of ARA in neutrophils and monocytes membranes in *Schizochytrium*-fed goats since DHA antagonizes ARA for the lipoxygenase and cyclooxygenase active sites [2]. The inhibition of the 5-LOX pathway can also indicate a suppression of LTB4 and LXA4, the principal pro-inflammatory leukotriene and lipoxin mediators, respectively [30]. Since this observation was most prominent in monocytes and the chemoattractant properties of the aforementioned mediators are well-known [30], the downregulation of chemotactic Interleukin-8 (*IL8*) expression in monocytes observed in our previous work [12] is now further validated. The LOX pathway is a well-documented pro-inflammatory route [30]. Moreover, both 12-LOX and 15-LOX can also catalyze the production of anti-inflammatory resolvins D (RvD) and protectins (PD) in the presence of DPA and DHA. In our study, the mRNA levels of *ALOX12* did not follow the inhibition trend of 5-LOX pathway; consequently, we can hypothesize that maresin (MaR), the prevailing anti-inflammatory mediator produced by the 12-LOX pathway was enhanced in *Schizochytrium*-fed goats (presence of DHA instead of ARA) compared to the CON (presence of ARA).

Prostanoids PGE2 and PGD2 are both pro-inflammatory and pro-resolving in self-limited inflammatory responses [30]. PGE2 and PGD2 are synthesized by the sequential actions of COX-1 (PTGS1) or COX-2 (PTGS2) followed by PGE2 synthase (PTGES) for PGE2 or PGD2 synthase (PTGDS) for PGD2 [30]. COX-1 and COX-2 are also involved in the first step of E-series resolvin (RvE) synthesis [30]. In this study, both the mRNA levels of *COX2* and *PTGER2* were downregulated in both monocytes and neutrophils in *Schizochytrium*-fed goats compared to the CON. The possible enhancement of DHA availability of cell membranes and its antagonisms with ARA may downregulate the expression of ARA-related prostanoid enzymes [2]. However, the overall pro-inflammatory inhibition may not be initiated by COX and LOX precursors as explained above, rather than the influence of another membrane receptor involved in the anti-inflammatory effects of ω3 FA namely G protein-coupled receptor 120 (GPR120) [31]. GPR120 regulates the TLR4 pathway either directly by LPS binding failure on TLRs, or indirectly via the inhibition of ΙΚΚβ kinase phosphorylation [31]. In addition, human B cells and macrophages incubated with DHA demonstrated a decreased expression of *TLR4*, while *GPR120* expression was upregulated [32]. In our previous study, the dietary supplementation with *Schizochytrium* spp. in goats inhibited the expression of TLR4-MYD88 pathway, resulting in the downregulation of *IL1B* [12]. In turn, *IL1B* can stimulate the expression of COX-2-dependent PGE2 synthesis [33].

In general, ω3 LCPUFAs seem to be well-studied downregulators of pro-inflammatory mediators and they are therefore considered to be potential immune-modulating feed components. However, ω6 DPA synthesized by *Schizochytrium* spp. biomass may have a stronger impact on prostanoids suppression compared to DHA. Nauroth et al. [34] reported that ω6 DPA suppresses the expression of both COX-2 and PGE2 in LPS-stimulated cells in vitro compared to DHA and EPA. These outcomes possess high biomolecular interest since ω6 DPA is an elongation product of ARA.

## 5. Conclusions

The high inclusion of *Schizochytrium* spp. in goats’ diet induced imbalances in the expression of genes regulating the antioxidant mechanisms of monocytes and neutrophils owed to NADPH oxidase activation. On the other hand, *Schizochytrium* spp. dietary supplementation suppressed the expression of genes that regulate the pro-inflammatory manifestation, with evidence suggesting an alternative route for eicosanoid production. Lastly, new insights were unveiled about the synergistic impact of DHA and ω6 DPA as immunoregulators.

## Figures and Tables

**Table 1:** Sequences, amplicon size, annealing temperature, and RefSeq number of primers used in real-time qPCR.

Gene	Sequence	Amplicon Size	Tm °C	GenBank RefSeq
*Glyceraldehyde-3-phosphate dehydrogenase (GAPDH)*	F: 5’-AAAGGCCATCACCATCTTCCA-3’	75	62	XM_005680968.3
	R: 5’-ACCACGTACTCAGCACCAGCAT-3’			
*Tyrosine 3-monooxygenase/tryptophan 5-monooxygenase activation protein zeta (YWHAZ)*	F: 5’-TGTTCTATTGTGCCTAGTACACTGT-3′	70	62	XM_018058314.1
	R: 5’-CATCAAGACTCACTGCCTCCC-3′			
*Catalase (CAT)*	F: 5′-GAGGAAACGCCTGTGTGAGA-3′	116	60	XM_005690077.3
	R: 5′-GGATGCGGGAGCCATATTCA-3′			
*Superoxide dismutase 1 (SOD1)*	F: 5′-ATCCACTTCGAGGCAAAGGG-3′	124	60	NM_001285550.1
	R: 5′-CTGCACTGGTACAGCCTTGT-3′			
*Superoxide dismutase 2 (SOD2)*	F: 5′-GCCCGATTATCTGAAGGCCA-3′	99	60	XM_018053428.1
	R: 5′-CTCAGTGTAAGGCTGACGGT-3′			
*Superoxide dismutase 3 (SOD3)*	F: 5′-CGAGTGTAAGGCCGTCTGAG-3′	76	60	XM_018049136.1
	R: 5′-GGACATAGAAGGGGTCTGCG-3′			
*Glutathione peroxidase 1 (GPX1)*	F: 5′-CATCGACATCGAGCCTGACA-3′	109	60	XM_005695962.3
	R: 5′-AAAATCCCCGGAGAGCAGTG-3′			
*Glutathione peroxidase 2 (GPX2)*	F: 5′-CCTCCCCACCCCTTTAATCG-3′	115	62	XM_005685982.3
	R: 5′-GGCTGATAGCACTGAGGTCG-3′			
*Glutathione peroxidase 3 (GPX3)*	F: 5′-GGAGGCCAAGGGGAAGTAAC-3′	114	60	XM_005683183.3
	R: 5′-GCATGGGAGTGTGGCATAGT-3′			
*Glutathione transferase 1 (MGST1)*	F: 5′-CTCCTGCTCGGATTCACACC-3′	88	60	XM_018048426.1
	R: 5′-TTAGGTGCGAAAGGTTGACCA-3′			
*Glutathione transferase 2 (MGST2)*	F: 5′-AAAGTTATGCCCCCATCCGT -3′	85	60	XM_013970519.2
	R:5′-CACCAGACCCAGACAAGTAGC -3′			
*Glutathione transferase 3 (MGST3)*	F: 5′-CCCCACTCTGATAGAGGCCA -3′	121	60	XM_013975063.2
	R:5′-GTAGTCGTCCAGCCTCGTTT -3′			
*Glutathione reductase (GSR)*	F: 5′-CTGCCCTGGGTTCTAAGACA-3′	104	60	XM_018041989.1
	R:5′-AGCATTCTCCAGCTCTTCGG-3′			
*Nicotinamide adenine dinucleotide phosphate oxidase 1; (NOX1)*	F: 5′-TCTTTCAAGCCTCGAGTCCC-3′	74	60	XM_018044365.1
	R: 5′-AGGTCCATGAAGCTCAGTGATG-3′			
*Nicotinamide adenine dinucleotide phosphate oxidase 2 (NOX2)*	F: 5′-ACGACCCAACTGGGATAACG-3′	127	60	XM_005700924.3
	R: 5′-GGAGTTGGAGATGCACTGCT-3′			
*Cyclooxygenase-2 (COX2)*	F: 5′-TCCCATCCATGCCAGAATCG-3′	77	60	XM_018060731.1
	R: 5′-CCTGTTCGGGTACAGTCACA-3′			
*Prostaglandin E receptor 2 (PTGER2)*	F: 5′-GGACACAAGCAGACCACGTA-3′	108	60	NM_001314255.1
	R: 5′-CATGCGGATGAGGTTGACGA-3′			
*Arachidonate 12-Lipoxygenase (ALOX12)*	F: 5′-AGGACTGCGCTCAAATCAGG-3′	83	60	XM_018064507.1
	R: 5′-TCCTGGAGAGTGGGCTTCTC-3′			
*Arachidonate 5-Lipoxygenase Activating Protein (ALOX5AP)*	F: 5′-ACTTTGTTGGCTACCTGGGG-3′	107	60	XM_005687536.3
	R: 5′-GTTGAGTATCCCAGCGAGGG-3′			
*Leukotriene A4 Hydrolase (LTA4H)*	F: 5′-TCCCTTTCTCTCGCGCTCAG-3′	78	61	XM_005680471.3
	R:5′-GTGAGGAGTCCCGATGCAC-3′			
*Leukotriene C4 Synthase (LTC4S)*	F: 5′-TGTCTAGGGCTGGAGGAAAG-3′	102	60	XM_018051605.1
	R: 5′-CAGAAGTACCAGGGAGCAGATG-3′			
*Cytosolic phospholipase A2 (PLA2G4A)*	F: 5′-TTGTGCTACAGAGAGGAGAGGA-3′	119	61	XM_018060732.1
	R: 5′-GTGCCACGTAGCACCACTAC-3′			

## Data Availability

The data are contained within the article and the Supplementary Materials.

## Supplementary Materials

The following supporting information can be downloaded at: https://www.mdpi.com/article/10.3390/antiox12020497/s1, Table S1. Chemical composition (g/Kg, as fed) and fatty acids profile (% of total fatty acid) of the diets.; Table S2. Mean and standard error of means (SEM) of relative transcript levels of several anti- and pro-oxidant-related and immune-related genes expressions in monocytes of goats fed the four experimental diets (CON, ALG20, ALG40 and ALG60) at three sampling time (20th, 40th and 60th).; Table S3. Mean and standard error of means (SEM) of relative transcript levels of several anti- and pro-oxidant-related and immune-related genes expressions in neutrophils of goats fed the four experimental diets (CON, ALG20, ALG40 and ALG60) at three sampling time (20th, 40th and 60th).
