# Supplementary material for: Schizochytrium spp. Dietary Supplementation Modulates Immune-Oxidative Transcriptional Signatures in Monocytes and Neutrophils of Dairy Goats
Source: Antioxidants (Basel). 2023 Feb 16;12(2):497. doi: 10.3390/antiox12020497 (PMC9952451; doi:10.3390/antiox12020497)
Supplement: Supplementary file 1 [file antioxidants-12-00497-s001.zip › antioxidants-2210349-supplementary.pdf]

Table S1. Chemical composition (g/Kg, as fed) and fatty acids profile (% of total fatty acid) of the diets.

**Chemical composition**	***CON***	***ALG20***	***ALG40***	***ALG60***	**Alfalfa hay**	**DHAgold™**
Dry matter	922	927	918	926	927	980
Crude protein	126	127	127	127	134	167
Ether extract	43	57	63	79	13	556
Crude fiber	50	52	52	56	319	45
NDF	157	152	149	169	503	-
ADF	61	56	57	64	381	-
Ash	92	95	93	89	72	88
**Fatty acid composition**						
$C_{14:0}$	0.1	1.4	2.8	3.7	2.3	5.9
$C_{16:0}$	14.3	15.2	17.1	18.7	31.6	13.1
$C_{18:0}$	3.9	3.2	2.7	2.5	5.8	0.3
$^{Cis\text{-}9}C_{18:1}$	36.1	29.8	24.2	21.8	11.6	n.d.
$C_{18:2\ n\text{-}6}$	42.2	36.5	31.1	28.6	26.4	n.d.
$C_{18:3\ n\text{-}3}$	1.7	1.6	1.3	1.2	19.7	0.1
$C_{22:5\ n\text{-}6}$	n.d.	2.9	5.3	6.2	n.d.	6.8
$C_{22:6\ n\text{-}3}$	0.1	7.3	13.4	15.5	n.d.	21.8

n.d.= no detectable; CON= control concentrate without experimental factors; ALG20= control concentrate with 20 g/ Kg *Schizochytrium spp.*; ALG40= control concentrate with 40 g/ Kg *Schizochytrium spp.*; ALG60= control concentrate with 60 g/ Kg *Schizochytrium spp.;* DHAgold= DSM feed industry (DSM Nutritional Products, Marousi, Greece); NDF= Neutral detergent fiber; ADF= Acid detergent fiber.

Table S2. Mean and standard error of means (SEM) of relative transcript levels of several anti- and pro-oxidant-related and immune-related genes expressions in monocytes of goats fed the four experimental diets (CON, ALG20, ALG40 and ALG60) at three sampling time (20th, 40th and 60th).

	DIET (D)					SAMPLING TIME (T)				EFFECT‡		
	CON	*ALG20*	*ALG40*	*ALG60*	SEM†	20	40	60	SEM†	D	T	DxT
CAT	0.224	0.217	0.219	0.240	0.012	0.190^{a}	0.226^{b}	0.259^{c}	0.005	0.562	0.001	0.559
MGST1	0.354	0.212	0.250	0.196	0.089	0.078^{a}	0.552^{b}	0.128^{c}	0.062	0.606	0.001	0.682
MGST2	0.025	0.020	0.022	0.020	0.007	0.009^{a}	0.045^{b}	0.011^{a}	0.004	0.961	0.002	0.971
MGST3	0.005	0.004	0.019	0.016	0.005	0.005^{a}	0.022^{b}	0.006^{a}	0.003	0.132	0.007	0.176
GSR	0.181	0.147	0.144	0.148	0.016	0.074^{a}	0.324^{b}	0.067^{a}	0.010	0.357	0.001	0.945
SOD1	1.408	1.597	1.603	1.823	0.165	0.877^{a}	3.085^{b}	0.861^{a}	0.145	0.390	0.001	0.505
SOD2	1.847^{a}	1.264^{ab}	1.269^{ab}	0.938^{b}	0.225	0.481^{a}	3.00^{b}	0.509^{a}	0.180	0.010	0.001	0.063
SOD3	0.0070^{a}	0.0003^{b}	0.0010^{b}	0.0030^{b}	0.002	0.002^{a}	0.006^{b}	0.001^{c}	0.001	0.044	0.016	0.105
GPX1	26.510	23.512	23.790	26.868	2.654	12.488^{a}	46.464^{b}	16.558^{a}	2.319	0.726	0.001	0.762
GPX2	0.0015	0.0014	0.0013	0.0014	0.0001	0.001^{a}	0.0023^{b}	0.001^{a}	0.0001	0.909	0.014	0.909
GPX3	0.252	0.049^{t}	0.068^{t}	0.278^{t}	0.086	0.099^{a}	0.345^{b}	0.040^{c}	0.078	0.099	0.013	0.328
NOX1	0.098^{t}	0.170	0.180^{t}	0.182^{t}	0.031	0.074^{a}	0.331^{b}	0.068^{a}	0.020	0.067	0.001	0.308
NOX2	5.294^{at}	8.376^{ab}	9.227^{b}	8.364^{tab}	1.279	3.612^{a}	16.192^{b}	3.642^{a}	0.618	0.042	0.001	0.158
COX2	1.103^{a}	0.670^{ab}	0.478^{b}	0.188^{b}	0.206	0.168^{a}	1.480^{b}	0.181^{a}	0.128	0.035	0.001	0.038
ALOX12	0.025	0.025	0.028	0.025	0.004	0.013^{a}	0.047^{b}	0.016^{a}	0.003	0.891	0.001	0.724
ALOX5AP	0.855^{a}	0.629^{ab}	0.435^{ab}	0.268^{b}	0.182	0.235^{a}	1.133^{b}	0.272^{a}	0.151	0.033	0.001	0.046
PLA2G4A	0.015	0.012	0.011	0.011	0.002	0.006^{a}	0.026^{b}	0.006^{a}	0.002	0.490	0.001	0.278
LTC4S	0.011	0.009	0.010	0.008	0.001	0.005^{a}	0.018^{b}	0.005^{a}	0.001	0.580	0.001	0.867
LTA4H	0.103^{ab}	0.082^{a}	0.092^{a}	0.122^{b}	0.008	0.058^{a}	0.176^{b}	0.065^{a}	0.008	0.015	0.001	0.867
PTGER2	0.456^{a}	0.204^{b}	0.207^{b}	0.120^{b}	0.060	0.020^{a}	0.071^{b}	0.010^{a}	0.035	0.004	0.001	0.001

Means with different superscript (a, b, c, d) between dietary treatments and (a, b, c) between sampling time differ significantly ($p \leq 0.05$) while, t ; trend refers to $p < 0.10$.
CON= control concentrate without microalgae; ALG20= control concentrate with 20 g/ Kg *Schizochytrium* spp.; ALG40= control concentrate with 40 g/ Kg *Schizochytrium* spp.; ALG60= control concentrate with 60 g/ Kg *Schizochytrium* spp.
‡ Effect: The dietary treatment (D), time (T), and the interaction between dietary treatment x time (DxT) effects were analyzed by ANOVA using a general linear model (GLM) for repeated measures and *Post hoc* analysis was performed when appropriate using LSD multiple range test. †SEM: Standard error of the meaN

Table S3. Mean and standard error of means (SEM) of relative transcript levels of several anti- and pro-oxidant-related and immune-related genes expressions in neutrophils of goats fed the four experimental diets (CON, ALG20, ALG40 and ALG60) at three sampling time (20th, 40th and 60th).

	DIET (D)					SAMPLING TIME (T)				EFFECT		
	CON	*ALG20*	*ALG40*	*ALG60*	SEM	20	40	60	SEM	D	T	DxT
CAT	0.171	0.210	0.171	0.190	0.016	0.164^{a}	0.187^{ab}	0.807^{b}	0.012	0.312	0.054	0.229
MGST1	0.413^{a}	0.334^{ab}	0.333^{ab}	0.281^{b}	0.040	0.269^{a}	0.415^{b}	0.358^{b}	0.029	0.037	0.001	0.413
MGST2	0.018	0.018	0.014	0.012	0.005	0.013	0.016	.017	0.003	0.697	0.374	0.527
MGST3	0.004	0.003	0.010	0.009	0.004	0.006	0.009	0.006	0.002	0.142	0.235	0.412
GSR	0.353	0.207	0.344	0.221	0.083	0.250	0.336	0.286	0.067	0.469	0.527	0.432
SOD1	0.418	0.458	0.339	0.424	0.050	0.393	0.403	0.433	0.031	0.405	0.505	0.618
SOD2	4.284	4.738	3.779	2.503	1.061	1.882^{a}	5.421^{b}	4.532^{b}	0.884	0.152	0.002	0.792
SOD3	0.0014	0.0018	0.0011	0.0011	0.0001	0.001	0.002	0.001	0.0001	0.391	0.292	0.651
GPX1	35.89	27.324	30.892	26.167	3.777	22.156^{a}	31.582^{b}	36.826^{b}	2.519	0.298	0.001	0.268
GPX2	0.010^{a}	0.004^{b}	0.007^{ab}	0.005^{b}	0.002	0.006^{a}	0.005^{b}	0.010^{b}	0.001	0.036	0.046	0.380
GPX3	0.167	0.213	0.146	0.137	0.058	0.159	0.195	0.148	0.036	0.793	0.729	0.719
NOX1	0.012^{a}	0.023^{ab}	0.013^{a}	0.025^{b}	0.003	0.015^{a}	0.024^{b}	0.018^{ab}	0.002	0.043	0.033	0.194
NOX2	1.993^{a}	2.49^{ab}	2.021^{a}	2.749^{b}	0.207	1.907^{a}	2.792^{b}	2.248^{ab}	0.156	0.045	0.001	0.071
COX2	0.433^{t}	0.356	0.274	0.245^{t}	0.084	0.258^{a}	0.439^{b}	0.258^{b}	0.051	0.066	0.029	0.749
ALOX12	0.016	0.015	0.013	0.012	0.002	0.010^{a}	0.015^{b}	0.017^{b}	0.002	0.595	0.001	0.858
ALOX5AP	2.107	1.964	1.589	1.374	0.379	1.456	2.188	1.754	0.289	0.409	0.113	0.599
PLA2G4A	0.008	0.008	0.008	0.009	0.001	0.011^{a}	0.008^{b}	0.005^{c}	0.001	0.936	0.001	0.584
LTC4S	0.007	0.004	0.006	0.005	0.002	0.008	0.004	0.005	0.001	0.760	0.281	0.114
LTA4H	0.124^{a}	0.071^{b}	0.102^{ab}	0.086^{ab}	0.016	0.078	0.094	0.115	0.012	0.028	0.107	0.715
PTGER2	0.756^{a}	0.467^{ab}	0.491^{ab}	0.329^{b}	0.123	0.316^{a}	0.687^{b}	0.506^{ab}	0.089	0.024	0.009	0.771

Means with different superscript (a, b, c, d) between dietary treatments and (a, b, c) between sampling time differ significantly ($p \leq 0.05$) while, t ; trend refers to $p < 0.10$.
CON= control concentrate without microalgae; ALG20= control concentrate with 20 g/ Kg *Schizochytrium* spp.; ALG40= control concentrate with 40 g/ Kg *Schizochytrium* spp.; ALG60= control concentrate with 60 g/ Kg *Schizochytrium* spp.
‡ Effect: The dietary treatment (D), time (T), and the interaction between dietary treatment x time (DxT) effects were analyzed by ANOVA using a general linear model (GLM) for repeated measures and *Post hoc* analysis was performed when appropriate using LSD multiple range test. †SEM: Standard error of the mean
